# Care during labor and birth for the prevention of intrapartum-related neonatal deaths: a systematic review and Delphi estimation of mortality effect

**DOI:** 10.1186/1471-2458-11-S3-S10

**Published:** 2011-04-13

**Authors:** Anne CC Lee, Simon Cousens, Gary L Darmstadt, Hannah Blencowe, Robert Pattinson, Neil F Moran, G Justus Hofmeyr, Rachel A Haws, Shereen Zulfiqar Bhutta, Joy E Lawn

**Affiliations:** 1 Johns Hopkins Bloomberg School of Public Health, Department of International Health, Baltimore MD, USA; 2 Department of Newborn Medicine, Brigham and Women’s Hospital, Boston, MA, USA; 3 London School of Tropical Medicine and Hygiene, London, UK; 4 Family Health Division, Global Health Program, Bill and Melinda Gates Foundation, Seattle WA, USA; 5 MRC Maternal and Infant Health Care Strategies Research unit, University of Pretoria, South Africa; 6 Grey’s Hospital, KwaZulu-Natal, South Africa; 7 University of the Witwatersrand and East London Hospital Complex, South Africa; 8 Jinnah Postgraduate Medical Center and the Aga Khan University, Karachi, Pakistan; 9 Saving Newborn Lives/Save the Children

## Abstract

**Background:**

Our objective was to estimate the effect of various childbirth care packages on neonatal mortality due to intrapartum-related events (“birth asphyxia”) in term babies for use in the Lives Saved Tool (LiST).

**Methods:**

We conducted a systematic literature review to identify studies or reviews of childbirth care packages as defined by United Nations norms (basic and comprehensive emergency obstetric care, skilled care at birth). We also reviewed Traditional Birth Attendant (TBA) training. Data were abstracted into standard tables and quality assessed by adapted GRADE criteria. For interventions with low quality evidence, but strong GRADE recommendation for implementation, an expert Delphi consensus process was conducted to estimate cause-specific mortality effects.

**Results:**

We identified evidence for the effect on perinatal/neonatal mortality of emergency obstetric care packages: 9 studies (8 observational, 1 quasi-experimental), and for skilled childbirth care: 10 studies (8 observational, 2 quasi-experimental). Studies were of low quality, but the GRADE recommendation for implementation is strong. Our Delphi process included 21 experts representing all WHO regions and achieved consensus on the reduction of intrapartum-related neonatal deaths by comprehensive emergency obstetric care (85%), basic emergency obstetric care (40%), and skilled birth care (25%). For TBA training we identified 2 meta-analyses and 9 studies reporting mortality effects (3 cRCT, 1 quasi-experimental, 5 observational). There was substantial between-study heterogeneity and the overall quality of evidence was low. Because the GRADE recommendation for TBA training is conditional on the context and region, the effect was not estimated through a Delphi or included in the LiST tool.

**Conclusion:**

Evidence quality is rated low, partly because of challenges in undertaking RCTs for obstetric interventions, which are considered standard of care. Additional challenges for evidence interpretation include varying definitions of obstetric packages and inconsistent measurement of mortality outcomes. Thus, the LiST effect estimates for skilled birth and emergency obstetric care were based on expert opinion. Using LiST modelling, universal coverage of comprehensive obstetric care could avert 591,000 intrapartum-related neonatal deaths each year. Investment in childbirth care packages should be a priority and accompanied by implementation research and further evaluation of intervention impact and cost.

**Funding:**

This work was supported by the Bill and Melinda Gates Foundation through a grant to the US Fund for UNICEF, and to Saving Newborn Lives Save the Children, through Save the Children US.

## Background

The remarkable decline in neonatal mortality rates in the middle of the 20th century in high income countries has been commonly credited to the advent of hygienic childbirth practices and modern obstetric care [1], with additional reductions since the 1970s attributed to increasingly intensive neonatal care. In low income countries, where skilled professionals attend fewer than half of deliveries, and each year 60 million births occur outside facilities [2], the burden of neonatal morbidity and mortality related to childbirth remains very high [3]. Intrapartum-related events in term babies associated with hypoxic injury (previously loosely termed “birth asphyxia”) are responsible for an estimated 814,000 neonatal deaths [4] and also one million stillbirths [5] each year, with perhaps one million disabled survivors with long-term neuro-developmental injury, including cerebral palsy, mental retardation, blindness, long term intellectual impairment and behavioral problems [6,7]. Childbirth is also the time of greatest risk for maternal deaths with at least 42% of the annual estimated 352,000 maternal deaths occurring during labor and the first 2 days after birth [3,8,9].

While skilled attendance at delivery and emergency obstetric care are the basis of modern obstetrics, there is remarkably limited impact evaluation. This gap is related both to methodological challenges such as the large sample sizes required for meaningful statistical comparisons, and also because many obstetric interventions were in routine practice before the advent of randomized controlled trials (RCTs), making it unethical, for example, to undertake a RCT of the impact of Caesarean section [10]. Estimates of the effectiveness of intrapartum care in reducing maternal and neonatal mortality and stillbirths are needed to inform healthcare planning and prioritization in low resource countries.

In this paper, we assess the evidence for effect on neonatal mortality of health service delivery packages during labor and childbirth. The terminology around childbirth care has been through various transitions in the last decade, and at times even different United Nations (UN) agencies use the same term differently [11]. Here, we have taken the latest UN consensus and reviewed the terminology for clarity (Table 1). ***Comprehensive emergency obstetric care* (*CEmOC*)** is the standard full package of obstetric care including Caesarean section and blood transfusion [12,13]. ***Basic emergency obstetric care* (*BEmOC*)** includes the six signal functions that should be available at first-level facilities which provide childbirth care: parenteral antibiotics, parenteral oxytoxics, parenteral anticonvulsants for pre-eclampsia or eclampsia, assisted vaginal delivery (including vacuum or forceps assistance for delivery, episiotomy, advanced skills for manual delivery of shoulder dystocia, skilled vaginal delivery of the breech infant), manual removal of the placenta, and removal of retained products [12-14]. ***Skilled childbirth care*** is defined by WHO as care provided by “an accredited health professional – such as a midwife, doctor or nurse – who has been educated and trained to proficiency in the skills needed to manage normal (uncomplicated) pregnancies, childbirth and the immediate postnatal period, and in the identification, management and referral of complications in women and newborns.” [12,13] For the purpose of these estimates, the effect of skilled attendance is considered as the attendant without additional obstetric care functions (BEmOC or CEmOC). We also reviewed the evidence for childbirth care by community cadres providing care at birth, such as a ***Traditional Birth Attendant* (*TBA*)**, defined by WHO as a person who “assists the mother during childbirth and who initially acquired her skills by delivering babies herself or though an apprenticeship to other TBAs” [15].

**Table 1:** Definitions of interventions and packages for care during labor and childbirth


***Comprehensive Emergency Obstetric Care*** **(*CEmOC*)**	Full package of CEmOC as per UN definitions [12,14], includes all six BEmOC functions PLUS: • Caesarean section • Blood transfusion

***Basic Emergency Obstetric Care*** **(*BEmOC*)**	UN definition of the 6 signal functions of BEmOC [12,14] • IV/IM antibiotics • IV/IM uterotonic drugs/oxytoxics • IV/IM anticonvulsants for pre-eclampsia and eclampsia (ie. magnesium sulfate) • Manual removal of placenta • Assisted vaginal delivery (episiotomy, instrumental delivery (forceps or vacuum extraction), advanced skills for manual delivery of shoulder dystocia, breech) • Removal of retained products (manual vacuum extraction, dilation and curettage) * Assuming no access to Caesarean section or blood transfusion

***Skilled childbirth care***	Skilled birth attendant defined by WHO, ICM, and FIGO as “an accredited health professional – such as a midwife, doctor or nurse – who has been educated and trained to proficiency in the skills needed to manage normal (uncomplicated) pregnancies, childbirth and the immediate postnatal period, and in the identification, management and referral of complications in women and newborns.” [13] The core intrapartum skills that should be provided include: • Clean delivery care • Monitoring onset and progress of labor with partograph • Monitoring maternal and fetal well-being during labor, identify maternal/fetal distress and taking appropriate action including referral • Manage normal vaginal delivery (including releasing a cord around the neck, delivery of shoulders, assisting a breech delivery) • Active management of third stage of labor • First line management of hemorrhage and hypertension in labor, referral as needed • Pain relief, hydration * For the purposes of this estimate assuming no access to instrumental delivery (forceps or vacuum extraction), Caesarean section or blood transfusion

***Trained Traditional Birth Attendant***	Traditional birth attendant defined by WHO as “a person who assists the mother during childbirth and who initially acquired her skilled by delivering babies herself or through an apprenticeship to other TBAs”[15]. A “trained TBA” is “any TBA who has received a short course of training through the modern health sector to upgrade her skills” [61]. TBAs may range from family members attending only occasional births to women with considerable expertise attending 20+ births/year. TBAs are not usually salaried, and typically not civil servants or employed by Ministry of Health.

**Timing of intervention and effect:** These packages include care provided during labor and birth, but in order to be effective, the care may have been initiated during the antenatal period (e.g., screening for abnormal lie and decision for elective Caesarean section, or screening and management of hypertensive disease of pregnancy/eclampsia). Some interventions are primarily intrapartum in timing such as management of acute intrapartum events including antepartum hemorrhage, cord prolapse and obstructed labor. **Not included in these effect estimates:** The effects on neonatal survival of specific interventions after birth for the baby are not included here as they are treated as single additional interventions in LiST and have been considered in detail in other reviews: - Stimulation and neonatal resuscitation at birth, - Postnatal healthy practices (breastfeeding, hygienic cord and skin care, thermal care). In addition, a few specific obstetric interventions which are in LiST but affect other neonatal causes of death have been considered in detail in other reviews including the following: - Corticosteroids for preterm labor (affects preterm deaths), - Antibiotics for preterm premature rupture of membranes (affects deaths from infections).	

Emergency obstetric care coverage remains extremely low, especially in rural areas: only 5% of births in rural South Asia and 1% in rural Sub-Saharan Africa are by Caesarean section [10]. Ensuring equitable coverage of skilled attendance may have been under resourced because it is considered complex and expensive [16]. If the impact of more complex childbirth care is high, then even given higher cost, the cost-effectiveness ratio may still be very favorable. There is a critical need for data regarding lives saved in order to inform investment choices and design effective programs. Skilled attendance coverage in Sub-Saharan Africa has increased little in the last decade. The Lives Saved Tool (LiST) has been designed to enable national (or sub-national) planning based on estimation of lives saved for mothers, neonates and children (http://www.jhsph.edu/dept/ih/IIP/list/index.html). The tool comes with a menu of interventions that are linked to mortality effects, and the user can increase coverage of each intervention from a baseline rate to compare the impact and cost of different interventions at varying levels of coverage.

## Objective

The objective of this review is to estimate the effect of different packages of care during labor and birth on intrapartum-related neonatal deaths in term babies, for inclusion in the Lives Saved Tool (LiST).

## Methods

We followed a standard approach to searches, abstraction and evaluation of evidence as set out by the Child Health Epidemiology Group (CHERG) for effect estimates to be used in the LiST model [17]. More details of the review methods, the adaptation of GRADE, the rules for attribution of mortality effect, and the LiST model, are published elsewhere [17,18].

### Searches for intervention evidence

**Figure 1:**
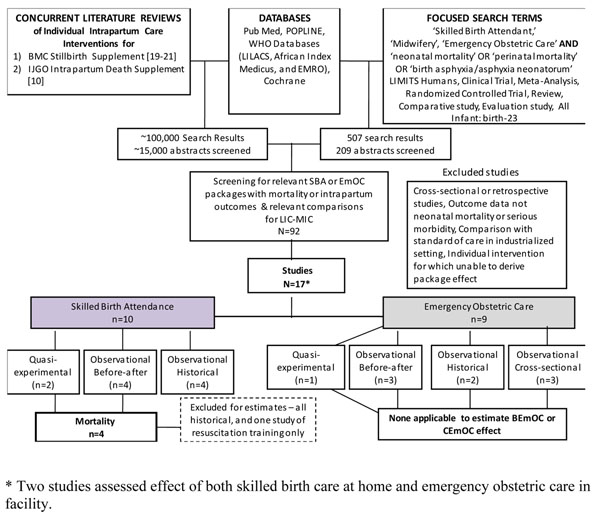
**Search strategies and results.** Skilled Birth Attendance and Emergency Obstetric Care and Intrapartum-Related Neonatal Deaths

**Figure 2:**
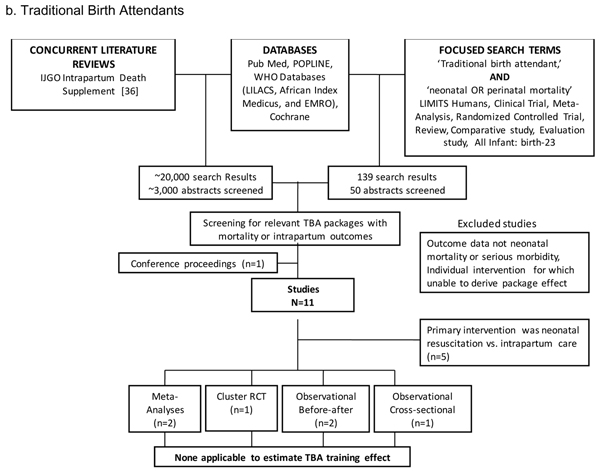
**Search strategies and results.** Traditional Birth Attendants

We undertook systematic searches of published literature from 1980 until March 2010. The original search was part of two parallel comprehensive literature reviews assessing the impact of intrapartum childbirth care on stillbirth [19-21] and intrapartum-related neonatal mortality [10]. The following databases were searched without language restrictions until March 2009: PubMed, POPLINE, Cochrane, EMRO, LILACS, and AIM (figures 1, 2). The search terms included MESH combinations of “skilled birth attendant,” “midwifery,” “basic/comprehensive emergency obstetric care,” “traditional birth attendant,” AND “birth asphyxia,” “asphyxia neonatorum,” or “neonatal-perinatal mortality.” A second updated search was conducted in March 2010 that required “skilled birth attendant,” “midwifery,” “emergency obstetric care,” “traditional birth attendant” AND “neonatal OR perinatal mortality.” Snowball searching, whereby literature referenced in key papers was included, was also employed.

### Inclusion/exclusion criteria

Data from studies meeting the inclusion criteria were extracted using a standard form (Additional File 1). We assessed the quality of each study using a standard approach developed by the Child Health Epidemiology Reference Group (CHERG) [17].

We applied the PICO format (Patient, Intervention, Comparison, and Outcome) to define the studies to be included as follows. The *population* of interest was pregnant women, or those in labor.

### Intervention definitions and those not considered in this review

The *interventions* considered are childbirth care packages and TBA training, as defined in Table 1. The study intervention was considered to meet package criteria [22] if 1) the authors directly described the intervention using package terminology (eg. BEmOC or CEmOC), or 2) the majority of package functions were reported to have been provided.

The effects of other interventions around childbirth are considered in separate reviews. While specific interventions, such as clean delivery practices and neonatal resuscitation, are considered essential elements of skilled birth attendance and emergency obstetric care, the effects are estimated separately in the LiST tool and reviewed in other papers [23,24] . The effects of individual childbirth interventions (such as fetal monitoring, partograph, labor induction, or Caesarean section), were reviewed separately in two concurrent supplement reviews published elsewhere and are not detailed again here [10,20,21]. In addition, those interventions specifically targeting the prevention of deaths due to preterm complications, even if provided during the intrapartum period, are not considered here, such as corticosteroids for prevention of preterm labor and antibiotics for preterm PROM) [25,26].

### Comparison group

In LiST the counterfactual is no care at all. Clearly a randomised trial with no skilled care provided at birth would be considered unethical, and most evaluations are non-randomised where the comparison is with standard practice. Hence we included studies with other comparison groups, such as before/after studies of improvements to existing services, cross-sectional and case-control studies, and historical data that reported mortality impact over several decades, recognizing that the majority of these studies did not control for confounders and were thus potentially subject to substantial bias.

### Outcome definitions

A *neonatal death* was defined as a death in the first 28 days of life, *early neonatal death* as death in the first 7 days of life, and *perinatal death* as a stillbirth (>1000 gms, > 28 weeks gestation) or death in the first 7 days of life. Deaths due to any cause are referred to as *all cause mortality* and *intrapartum-related neonatal death* classifies babies who die from childbirth related hypoxic events, (ie. what was previously referred to as “birth asphyxia”). While the term “birth asphyxia” has been used to describe babies who do not breathe at birth, the term is no longer recommended for epidemiological use in cause-of-death attribution [5,27]. *Intrapartum-related neonatal mortality* is defined by CHERG, based on ICD 10 rules and recent global consensus statements, as term babies who die after neonatal encephalopathy, or death prior to onset of neonatal encephalopathy, with evidence of intrapartum injury or acute intrapartum events [5,27]. *Neonatal encephalopathy* (*NE*) may directly result from intrapartum hypoxia and is considered a predictive marker of long term morbidity and mortality [3]. NE is defined as a “disturbance of neurological function in the earliest days of life in the term infant manifested by difficulty initiating and maintaining respiration, depression of tone and reflexes, abnormal level of consciousness and often by seizures [28,29].” *Hypoxic Ischemic Encephalopathy* is the condition of neonatal encephalopathy following severe hypoxic injury, however, is not recommended unless there is clear evidence of sufficient hypoxemia to account for impaired brain function [30].

We also examined studies that reported all cause neonatal mortality or specific morbidity, notably NE. We did not examine Apgar score as an outcome since our goal was to establish mortality effect estimates and the Apgar score is considered to be an unreliable indicator of mortality [31]. The effects of intrapartum care on stillbirths and maternal outcomes are also important and are reviewed elsewhere in this supplement [32].

### Ecologic analysis of variation in neonatal encephalopathy incidence

**Figure 3:**
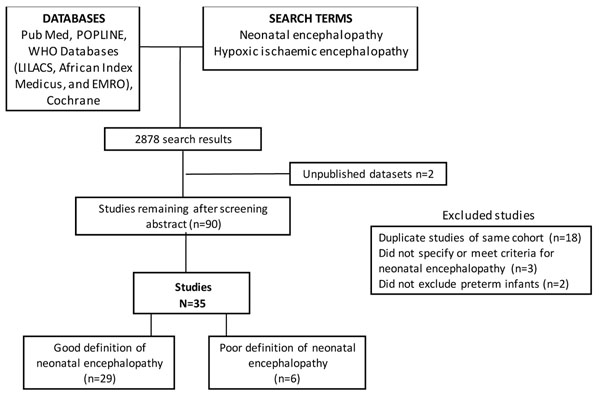
**Search strategies and results.** Incidence of neonatal encephalopathy

Given the paucity of direct evidence of package impact, we also conducted an ecological analysis to examine the relationship between NE incidence and coverage of childbirth care, drawing on a systematic review for the Global Burden of Disease Project, undertaken with the Child Health Epidemiology Reference Group [33]. In brief, PubMed, POPLINE, Cochrane, EMRO, EMBASE, LILACS, and AIM databases were searched using the terms “neonatal encephalopathy” and “hypoxic ischemic/ischemic encephalopathy” (figure 3). All titles were reviewed and articles were retrieved that had data on incidence, case fatality rates or chronic impairment. Potentially relevant country covariates, including % skilled attendance, % facility delivery, and % Caesarean section, were obtained from UN databases [2]. The natural log of the neonatal encephalopathy incidence rate was regressed on each obstetric indicator of interest using simple linear regression.

### Delphi Process for establishing expert consensus

For interventions with low or very low quality evidence but strong recommendation for program implementation [34,35], as per CHERG rules for LiST effect estimates, we sought expert consensus via the Delphi method [36]. We invited a panel of experts in obstetrics, gynecology and newborn health from all WHO regions and including multiple disciplines - program management, research, clinical obstetrics, and general paediatrics. The questionnaire was developed by JL, ACL, NM and GLD through several rounds of pilot testing. The survey was sent by email and included the background and aims of the Delphi process, evidence identified, and requested seven different effect estimates (Additional File 2). Respondents were allowed the option of anonymous response. The median response and range were determined for each question. Consensus was defined *a priori* as having been achieved when the inter-quartile range of responses to a given question was < 30%. For those estimates not reaching consensus on the first round, the results were electronically distributed to the panel, virtual discussion allowed, and a second round of email questionnaires sent.

### Analyses and summary measures

We conducted meta-analyses for mortality outcomes [neonatal mortality rate (NMR), perinatal mortality rate (PNMR), and early neonatal mortality rate (ENMR)] of observational before-after studies of community-based skilled birth attendants. Studies were considered for inclusion in the meta-analysis that had comparable intervention, study design, and outcome of interest. Statistical analyses were performed using STATA 10.0. The Mantel-Haenszel pooled risk ratio (RR)—or where there was evidence of heterogeneity (p<0.05), the DerSimonian-Laird pooled RR—and 95% confidence interval (CI) were calculated. For the Delphi panels, expert estimates were entered in an Excel spreadsheet and simple descriptive statistics were produced.

## Results

The search strategies and results are summarised in figures 1, 2. From the combined searches for skilled birth attendance and emergency obstetric care, which yielded around 15,000 abstracts, we retrieved 92 papers, reports or conference abstracts for full text review. From these, 17 studies reporting outcomes and comparisons of interest were identified. For the combined searches for traditional birth attendants, a total of around 3000 abstracts were identified, yielding 11 articles of interest.

### Results of literature review

#### Emergency obstetric care

Overall, few studies presented comparisons of childbirth care packages consistent with the UN definitions (table 1). The 9 studies of emergency obstetric care packages reporting neonatal mortality outcomes identified for this review were low quality and heterogeneous in terms of intervention content (Table 2), and not suitable for meta-analysis or for the LiST mortality effect estimation.

**Table 2:** Studies of the effect of Basic or Comprehensive Emergency Obstetric Care on perinatal-neonatal mortality or intrapartum-related outcomes

Author	Study Years	Setting	Study Design	Intervention definition	Concurrent interventions	Intervention Coverage	Total Births A) Endline B) Baseline	Outcomes	Effect on outcome RR/OR (95% CI)
Ronsmans 2010[37]	1987-2005	Matlab, Bangladesh	Observational cross-sectional	1987-1996: skilled home birth care w/midwives providing antenatal care, basic obstetric care (labor monitoring), essential newborn care; 1996 onwards facility based birth with BEmOC (partograph, active management 3^rd ^stage, antibiotics, management preeclampsia). Highest level care received (BEmOC, CEmOC, vs no skilled care)	Antepartum care, Essential newborn care, Strengthening of referral and transport systems	CEmOC 0.5% in 1987 to 11.7% in 2005 BEmOC 4.7% in 1987 to 40.9% in 2005	CEmOC 3084; BEmOC 9954; No skilled Care 40177	1) ENMR 2) Stillbirth	1)CEmOC aOR 2.69 (2.16-3.37) BEmOC aOR 1.47 (1.27-3.37) 2) CEmOC aOR 6.61(5.62-7.79) BEmOC aOR 1.51(1.31-1.73)

Berglund 2010[44]	2003-2004	3 Maternity Hospitals; Ukraine	Observational before-after	Training all maternity staff (obstetricians, neonataologists, midwives, anesthesiologists) in 2 week WHO "Effective Perinatal Care" program, including use of partogram, emergency obstetric and neonatal care (resuscitation).	Anesthesia; neonatal resuscitation & special care, thermoregulation	All maternity staff in 3 hospitals	A) 1696 B) 2439	1) ENMR	No significant effect

Hounton 2008[38,39,52]	2001-2005	Rural Ouargaye and Diapaga districts, Burkina Faso	Quasi-experimental	Upgrading of hospital, health centers in intervention area. Mid-level, referral facilities: emergency obstetric care training. First-level centers: training in prevention of complications and early detection -referral for emergencies. Quality improvement infrastructure upgrading, equipment and supplies	National policies and guidelines; Mobilising/educating communities to plan for and use maternal health services	Training in 1 district hospital and 13/19 health centers	18,658 births intervention district 2004-5; 21,788 births comparison district 2004-5	1) PMR	1) OR 0.75(0.70-0.80)

Draycott 2006 [41]	1998-2003	South Mead Hospital, UK	Before-after	EOC training course: CTG interpretation, course of action, obstetric emergency drills (dystocia, PPH, eclampsia, twins, breech, resuscitation)		Mandatory course for all midwives	A) 11030 B) 8430	1) HIE (MacLennan):	1) RR 0.50(0.26-0.95)

Edmond 2002[42]	1995-1998	Natal, Northeast Brazil	Observational before-after	Opening of primary maternity facilities at polyclinic to serve low risk deliveries in the community. Pre-booking of deliveries of high risk pregnancies at Maternity hospital with CEmOC capacity.	ANC, community health agents training in community health clinics	Deliveries at maternity clinics increased from 0% to 51%	A) 536 B) 679	1) ENMR 2) Stillbirth 3) PMR	1) RR 0.12 (0.04-0.40) 2) RR 0.66 (0.47-0.94) 3) RR 0.52 (0.37-0.73)

McCord 2001[43]	1996-1999	Rural Maharashtra, India	Cross-sectional	Comparison of perinatal mortality among births occurring at home vs. in hospital, some with CEmOC		85% home births, 15% in hospital.	Home: 2436 Hospital: 425	1) PMR	PMR 27.1 (home births) vs 87 (hospital deliveries)

Koblinsky 1999[40]	1957-1990s	Malaysia	Historical-ecological	1960 s Training of professional village midwives, linking to regional clinics, referral to district hospitals; 1980's shift to facility births with BEmOC	3 decades of perinatal care and obstetric care upgrading	95% of births by midwives (1996); 80% of risk deliveries in hospital (1998)	NS	1) NMR	NMR from 75.5 (1957) to 14.8 (1991)

Korhonen 1994[45]	1986-1991	Helsinki, Finland	Cross-sectional	Emergency Caesarean Team in Hospital vs. On call (out of hospital, 10 minute average delay)		NS	60 in hospital; 41 on call	1) Fetal Death; 2) HIE	3 in utero fetal deaths and 1 HIE in control (on-call) group vs 0 hospital

Piekkala 1985[1]	1968-1982	University Hospital, Turku Finland	Historical	15 year improvement in obstetric management: Cesearean rate increase from 4-12%; vaginal breech delivery from 4 to 1%; implementation of antepartum CTG (monitoring increase from 0 to 90%)	Corticosteroids, Neonatal intensive care, respiratory therapy, fluid-nutritional therapy	Referral hospital for 10% of population	A) 5,410 B) 5,996	1) PMR 2) Intrapartum mortality	1) RR 0.39 2) RR 0.29

We identified one study that compared ***basic*** and ***comprehensive emergency obstetric care*** with no skilled care with respect to neonatal mortality outcomes [37]. Ronsmans and colleagues analyzed health and demographic surveillance system data from 1987-2005 in Matlab, Bangladesh to examine the relationship between the use of BEmOC and CEmOC with early neonatal mortality and stillbirth [37]. They found that women receiving BEmOC and CEmOC had a higher risk of early neonatal mortality (BEmOC aOR 1.47, 95% CI 1.27-1.69; CEmOC aOR 2.69, 95% CI 2.16-3.37) compared to mothers delivering at home without skilled care. However this observational study is prone to selection bias, as skilled care/emergency obstetric care was likely sought for higher-risk, complicated deliveries, and thus the observed association is unlikely to reflect the population effect of the intervention [37].

The Skilled Care Initiative in Burkina Faso involved multiple activities to increase access to skilled birth care, including improving availability and quality of CEmOC by upgrading hospital capacity, equipment, and training in CEmOC at the district hospital (Table 2) [38,39]. At the end of the intervention period the PMR was 27.5/1000 in the intervention district compared with 33/1000 in the control district (OR 0.75, 95% CI 0.70-0.80) [38]. However, it is unclear how similar PMRs were in the intervention and control districts at the beginning of the intervention, and CEmOC was just one component of a complex intervention that also included community mobilization and education.

We identified historical reports from Malaysia [40] and Finland [1] that reported NMR trends coinciding with improvements in obstetric and neonatal care. In Malaysia, over three decades (1960-1990s), a national strategy to increase skilled birth attendance was implemented which included training professional village midwives (1970-80s), establishing links with district and referral hospitals, and a gradual shift to births in facilities with capacity for basic emergency obstetric care (1985-1990s). By 1995, institutional delivery had increased to 88% and the national NMR had declined from 75.5 in 1957 to 14.8 in 1991 [40]. In a Finnish university hospital, multiple obstetric and neonatal care improvements were instituted from 1968-1982 (including increased intrapartum monitoring, Caesarean section, corticosteroid therapy, amniotic fluid surfactant determination, and reduction in vaginal breech deliveries). Over the same time period, a 71% reduction in intrapartum-related neonatal mortality and a 61% reduction in all-cause perinatal mortality was observed. However, the effect of improved neonatal intensive care is likely to have played a major additional role in this mortality reduction.

In a tertiary care hospital in the UK, following an EmOC training course (cardiotocography interpretation; emergency drills for dystocia postpartum hemorrhage, eclampsia, breech delivery, and neonatal resuscitation) for obstetricians and midwives, a 50% reduction in hypoxic ischemic encephalopathy (95% CI: 0.26-0.95) was observed [41]. However, baseline care was likely substantially more complex than in the ‘average’ low-income country setting, and thus, this may underestimate the effect compared with no care. In addition the observed mortality reduction includes the effect of training in neonatal resuscitation, which is a separate intervention in LiST. Additional studies which provide supporting evidence of package effect are shown in Table 2, [42-45].

**Figure 4:**
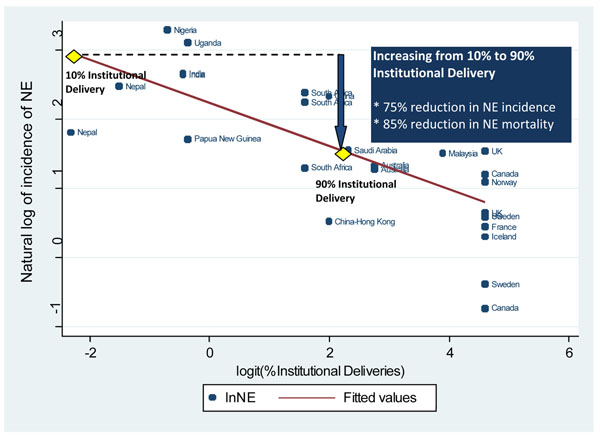
**Variation of the incidence of neonatal encephalopathy (NE) with the natural log of the proportion of institutional deliveries**. Legend: Each dot represents NE incidence data reported by a single study. For some countries more than one incidence was reported. The regression line is modeled as: lnNE=2.237 – 0.311 * logit (% Institutional Delivery) R^2^=0.50 According to this model, when increasing from settings with very low proportion of births in facilities (10%) to settings with high proportions of facility deliveries (90%), the incidence of neonatal encephalopathy decreases by 75%. When applying case fatality rates for neonatal encephalopathy based on the respective mortality setting, mortality from neonatal encephalopathy is reduced by 85% when facility birth is increased from 10% to 90%.

Our ecological analysis of the association between NE incidence and the proportion of institutional births is shown in Figure 4. The modelled incidence of neonatal encephalopathy when 10% of deliveries take place in health facilities was 18.6/1000 live births. Given a neonatal case fatality ratio of 25% using the median neonatal case fatality in high mortality level settings (NMR>15) from the literature review [33], the neonatal encephalopathy mortality rate would be around 4.7/1000 live births. When 90% of births take place in a facility, the modelled incidence of neonatal encephalopathy is 4.7/1000 live births (figure 4). Given a case fatality ratio of 15% [33], this results in a neonatal encephalopathy mortality rate of 0.7/1000 live births, which is around the reported rate for associated obstetric factors in the UK[46]. Thus, comparing 10% facility birth and 90% facility births, there is approximately a 75% reduction in the incidence of neonatal encephalopathy and an 85% reduction in neonatal encephalopathy-related mortality. This reduction, however, assumes that facility birth equates to prompt access to emergency obstetric care, and includes the effect of neonatal resuscitation and ongoing facility-based neonatal care, both of which may not be available in many facilities in low-resource settings. Hence this effect size (85%) may be expected to be above the upper limit of the effect of comprehensive obstetric care, not including resuscitation or ongoing neonatal care.

### Skilled childbirth care

For a delivery attendant alone, provider training may avert hypoxic brain injury by *primary prevention* via early recognition and referral for childbirth complications, or by *secondary prevention*, via managing the non-breathing baby with essential newborn care and neonatal resuscitation. The focus of this current review is on primary prevention, as neonatal resuscitation and thermal care are reviewed separately for LiST [24,47]. The evidence with respect to home-based skilled childbirth care has been reviewed in detail elsewhere [35]. We identified 10 studies reporting the impact of community-based skilled birth attendants on intrapartum-related perinatal or neonatal mortality (Tables 3 and 4): 2 quasi-experimental studies, 4 before-after studies, and 4 observational historical studies. Nine studies were from low- or middle-income settings.

**Table 3:** Studies of the impact of community skilled birth attendants on perinatal-neonatal mortality

Author	Study Years	Country	Setting	Study Design	Primary Intervention	Concurrent Interventions	Intervention Coverage	Total N A) Intervention B) Comparison	Outcomes Measured	Effect on outcome (95% CI)
Ronsmans 2008[50]	1975-1999	Matlab, Bangladesh	Rural, 1987-1996 SBA at home	Quasi-experimental (†use of before-after data in pooled anlaysis)	Posting of midwives in villages to increase skilled home birth (antenatal, basic obstetric, care including labor monitoring, essential newborn care) until 1996. After 1996, facility based strategy with upgrading of health centers in basic obstetric care (partograph use, active management 3^rd ^stage, antibiotics, magnesium)	Strengthening referral systems, Transport to BEMOC or CEmOC	25% of births attended by SBA during home birth period	A) 19085 (ICDDR,B 1989-1995) B) 22821 (ICDDR,B 1982-1988)	1) IPR-NMR 2) NMR † 3) ENMR† 4) PMR†	1) 0.78 (NS) 2) 0.83 (0.76-0.91) 3) 0.89 (0.80-0.97) 4) 0.92 (0.84-0.98)

Yan 1989[48]	1983-1986	Shunyi, China	Rural Shunyi County, 7 of 29 townships	Before-after	Village doctors-midwives identify risk and either manage (external cephalic version, blood pressure monitoring) or refer mothers to county hospital	Improvement of neonatal ward in county hospital	96% of pregnant women seen by village doctor-midwife	A) 2335 B) 2212	1) PMR 2) EMR 3) IP-PMR	1) 0.66 (0.44-0.98) 2) 0.77 (0.43-1.36) 3) 0.73 (*)

Ibrahim 1992[49]	1985-1988	Khartoum, Sudan	Rural, 91% home delivery	Before-after	Training and upgrading of skills of village midwives (antenatal care, monitoring in labor)	Data collection maternal-perinatal outcomes, referral system to hospital	91% of births delivered by village midwives	A) 2298 B) 3977	1) NMR 2) ENMR 3) SBR	1) 0.68 (0.48-0.97) 2) 0.78 (0.61-1.01) 3) 0.85 (0.60-1.19)

Alisjahbana 1995[51]	1992-1993	West Java, Indonesia	Rural villages, West Java; Tanjungsari district	Quasi-experimental (use of before-after data in pooled analysis)	Training physicians and village midwives on danger signs, case management in pregnancy, labor, delivery, postpartum; development of birthing homes	Training TBAs in pregnancy detection, complications and referral; communications and transportation	92% of births with professional provider	A) 1176 B) 1099	1) PMR	0.75 (0.51-1.10)

**Table 4:** Studies of the impact of community skilled birth attendants on perinatal-neonatal mortality, excluded from meta-analysis

Author	Study Years	Country	Setting	Study Design	Primary Intervention	Concurrent Interventions	Intervention Coverage	Total N A) Intervention B) Comparison	Outcome Measured	Effect on outcome RR/OR (95% CI)
Matthews 2004[59]	1999-2002	Ghana	Rural Brong Ahafo district	Before-after	Training midwives in health facilities on use of partograph and emergency obstetric skills	TBA Training in danger signs, Emergency obstetric transport service	NS	A) 768 B) 575	1) PMR	NS

Andersson 2000[55]	1831-1899	Sweden	18 Parishes Northern Sweden	Historical	1829 Training of midwives in use of forceps, "sharp hooks and perforators"	1881 antiseptic techniques	73% of home deliveries attended by midwives at endline (43% baseline)	NS	1) PMR	1) 0.71(0.62-0.82)

Hatt 2009[56]	1986-2002	Indonesia	National DHS Data	Historical	Village midwife training program started in 1989, by 1995 50,000 trained. In 1996 competency based training, neonatal resuscitation	2 decades of national perinatal care and obstetric care upgrading	Proportion of deliveries attended by midwives increased from 12% (1986) to 30% (2002)	NS	1) ENMR 2) First day mortality	1) 0.97 (0.95-0.99) per year reduction 2) 0.98(0.95-1.02) per year reduction

Koblinsky 1999[40]	1957-1990s	Malaysia	National NMR	Historical-ecological	1960 s Training of professional village midwives, linking to regional clinics, referral to district hospitals; 1980's shift to facility births	3 decades of perinatal care and obstetric care upgrading	By 1986, 95% of home births by midwives; by 1995, 88% institutional delivery; 90% of women with high risk, 80% moderate risk delivering in hospitals	NS	1) NMR	NMR from 75.5 (1957) to 14.8 (1991)

PATH 2006[58]	2003-2004	Cirebon, Indonesia	Rural Cirebon district, west Java, pop 2 mill	Before-After	Training mid-wives in management of labor, birth asphyxia, tube-mask resuscitation, refresher training/supervision		60% of asphyxia cases managed by midwives. Uncertain coverage	Est 44000	1) IPR-NMR 2) NMR 3) SBR	1) 0.39 (0.31- 0.48) 2) 0.60 (0.53-0.68) 3) 0.39 (0.31-0.48)

Shankar 2008[57]	1989-2003	Indonesia	National NMR	Historical	Village midwife training program started in 1989, by 1995 50,000 trained. In 1996 competency based training program including neonatal resuscitation	2 decades of national perinatal care and obstetric care upgrading	In rural areas skilled attendance increased from 22% to 55%	NS	1) NMR	NMR decreased from 32 to 20/1000 over 14 years

NS = Not stated in article

Four studies met our inclusion criteria and had trained community midwives [48-51] or village doctors [48] in intrapartum monitoring and management, with appropriate links to the health system, including referral and or transport to BEmOC or CEmOC facilities. Additional file 1 and Table 5 shows the GRADE table of included studies and their limitations. Only one study reported the effect of training community midwives on *intrapartum-related neonatal mortality* (RR 0.78, 95% CI 0.64-0.95) [50].

**Table 5:** GRADE summary table for the impact of community skilled birth attendants on perinatal-neonatal outcomes

	Study Quality					Summary of Findings				
				**Directness**		**Endline**		**Baseline**		

**No of studies**	**Design**	**Limitations**	**Consistency**	**Generalizability to Population of Interest**	**Generalizability to intervention of interest**	**Events**	**Births**	**Events**	**Births**	**Relative Risk (95% CI)**

***Neonatal Mortality*** **(*****Intrapartum-related*****)** ***: Low outcome specific quality***										

1 [50]	Quasi-experimental	Several interventions simultaneously and changes also in comparison villages		Community-setting LIC-MIC, South Asia	Yes	NS	19,085	NS	22,413	0.78 (0.64-0.95)

***Neonatal Mortality*** **(*****All Cause*****)** ***: Low outcome specific quality***										

2[49,50]	Observational, before-after	Low quality, before-after comparisons	No evidence of heterogeneity (p=0.28)	Community-setting LIC-MIC	Yes	794	21383	1186	26798	0.82 (0.75-0.90)^a^

***Early Neonatal Mortality*** **(*****All Cause*****)** ***: Low outcome specific quality***										

3 [48-50]	Observational, before-after	Low quality, before-after comparisons	No evidence of heterogeneity (p=0.50)	Community-setting LIC-MIC	Yes	597	23718	837	29010	0.87 (0.79-0.97)^a^

***Perinatal Mortality*** **(*****All Cause*****)** ***: Low outcome specific quality***										

4 [48-51]	Observational, before-after	Low quality, before-after comparisons	Evidence of heterogeneity (p=0.12)	Community-setting LIC-MIC	Yes	670	21981	909	27621	0.88 (0.83-.95)^b^

NS= Not Stated

a) MH pooled RR; b) D & L pooled RR random effect meta-analysis

**Figure 5:**
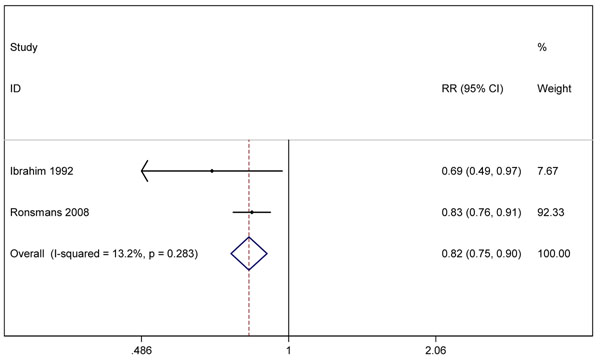
Meta-analysis of effect of skilled birth attendance in the community on neonatal or perinatal outcomes (Effect on all cause Neonatal Mortality Rate)

**Figure 6:**
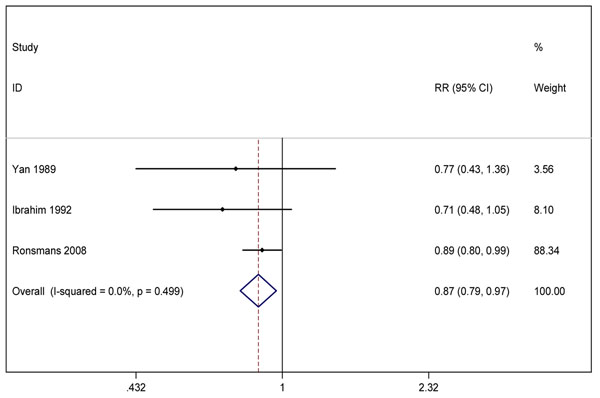
Meta-analysis of effect of skilled birth attendance in the community on neonatal or perinatal outcomes (Effect on Early Neonatal Mortality Rate)

**Figure 7:**
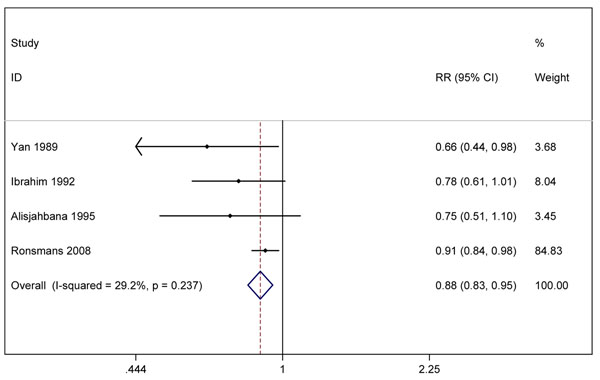
Meta-analysis of effect of skilled birth attendance in the community on neonatal or perinatal outcomes (Effect on Perinatal Mortality Rate)

We undertook meta-analysis for three outcomes (figures 5, 6, 7). The before-after data was used instead of the quasi-experimental comparisons because in one study the control group had different baseline characteristics [52], and in the other, there was contamination of the intervention in the comparison areas [50]. Two studies [49,50] reported the effect on *all-cause neonatal mortality* (pooled effect size RR 0.82, 95% CI 0.75-0.90). Three studies [48-50] reported the effects on *early neonatal mortality* (pooled effect size RR 0.87, 95% CI 0.79-0.97), which is more reflective of intrapartum-related mortality than all-cause NMR given that ~90% of “asphyxia” deaths occur in the first week of life [53,54]. Four studies reported the effect on *perinatal mortality*; the pooled effect was RR 0.88 (95% CI 0.83-0.95) [48-51]. While the data appear to indicate a consistent small protective effect of skilled childbirth care and all of the studies were conducted in low-income countries, the overall quality of the evidence is low by GRADE criteria [17].

Six studies of community midwives were excluded from these meta-analyses. Of these, four historical studies were excluded due to the very low data quality [40,55-57] . We also excluded a study from PATH Indonesia [58], which was a before-after design that did not accurately determine the denominator of live births and was primarily focused on training for neonatal resuscitation. The Matthews study [59] was excluded as the midwives and EmOC skills training were facility-based, while the community based component involved TBA training only.

### Traditional birth attendant training

The intervention reviewed is the impact of training TBAs in childbirth care, or primary prevention via early recognition and referral for obstetric emergencies, and excludes neonatal resuscitation, which is reviewed separately. The evidence for TBA training has been reviewed in detail elsewhere [35]. We present here a summary of the main findings. We identified one review [60], later adapted as a Cochrane [61], and 9 studies of TBAs with neonatal mortality outcomes (figure 2). Of the 9 studies, 5 studies were excluded as they focused primarily on neonatal resuscitation training versus primary prevention leaving 1 cluster RCT, 2 before-after studies and 1 cross-sectional study of interest (table 6).

**Table 6:** Individual studies of the effect of traditional birth attendant training in intrapartum care on perinatal-neonatal mortality

Author	Study years	Setting	Study Design	Intervention definition	Concurrent interventions	Intervention Coverage	Total N (A=intervention/endline; B=control/baseline)	Outcomes	Effect on outcome RR/OR (95% CI)
O’Rourke[66]	1991	Rural Guatemala	Before-after comparison	3-month hospital-based training program for TBAs - identification of obstetric emergency and referral; encouragement to attend hospital deliveries; strengthening relationships between TBAs and hospital staff		Studied only those patients who were sucessfully referred	A) 465; B) 39	1) PMR among referred infants*	RR 0.73

Greenwood et al. [86]	1983	Rural Gambia	Before-after comparison	TBA training in intervention villages within a comprehensive primary care program; 10 week training courseantenatal-postnatal care, referral signs; distribute clean birth kit and malaria prophylaxis	Introduction of comprehensive primary health care program, transport improvements	65%	A) 1159 B) 659	1) NMR; 2) PMR	1) RR 0.66; 2) RR 0.92

Janowitz et al. [74]	1984-85	Rural NE Brazil	Cross-sectional	TBA training especially in recognition of childbirth complications and referral. Non-randomized comparison of trained TBAs with high case load (>29 births per year) versus unattended home births	Establishment of “mini- maternities” with telephones for TBA births.	55%	A) 906; B) 118	1) NMR	RR 0.60

Jokhio et al. [65]	1998	Rural Pakistan, Larkana,	Cluster RCT	TBA training in antepartum, intrapartum, postpartum, and neonatal care; distribution of clean delivery kits; referral for emergency obstetrical care.	Lady health workers also trained to support TBA and link community-health center services.	74%	A) 10114; B) 9443	1) PMR; 2) NMR; 3) SBR	1) aOR 0.71 (0.62-0.83); 2) aOR 0.70 (0.59-0.82); 3) aOR 0.69 (0.57-0.83)

**Excluded from present review --Primary intervention was neonatal resuscitation**									

Carlo et al[68].	2005-2007	Argentina, DR Congo, Guatamala, India, Pakistan, Zambia	Before-after study	training of community birth attendants (TBAs, nurses) in WHO Essential Newborn Care , including basic resuscitation with bag-mask in 6 countries	Clean delivery, thermal protection, breastfeeding, kangaroo care	78% of births (post)	A) 22,626; B) 35,017	1) PMR; 2) SBR; 3) ENMR	1) RR 0.85 (0.70-1.02); 2) RR 0.69 (0.54-0.88); 3) RR 0.99 (0.81-1.22)

Kumar et al[63]	ns	Rural India	Quasi-experimental	TBAs trained in "advanced" resuscitation with suction and bag-mask vs. usual mouth-mouth resuscitation		TBAs delivered 92% of babies at home	A) 964; B) 884	1) "asphyxia" mortality; 2) PMR	1) RR 0.30 (0.11-0.81); 2) RR 0.82 (0.56-1.19)

Daga et al[87]	1988	Rural India	Before-after	TBA training in basic mouth-to -mouth breathing	Management of low birth weight, hypothermia; transport and referral of high risk babies to hospital	90%	A) 321; B) 660	1) PMR; 2) NMR; 3) SBR	1) RR 0.59 (0.32-1.09); 2) RR 0.39 (0.21-0.69); 3) RR 0.49 (0.16, 1.50)

Gill et al[67]	2006	Rural Zambia	Cluster RCT	Training of TBAs in a modified neonatal resuscitation program (NRP) w/resuscitator facemask	prevention of hypothermia, antibiotic treatment and facilitated referral for presumptive neonatal sepsis	uncertain	A) 2007 B) 1552	1) NMR; 2) “asphyxia” mortality	1) aRR 0.55 (0.33-0.90); 2) aRR 0.37(0.17-0.81)

Azad et al [88]	2004	Rural Bangladesh	Cluster RCT, factorial design	Intervention arm: Training of TBAs in neonatal resuscitation with bag-valve mask, with subsequent retraining; Control arm: Training of TBAs in mouth-to-mouth resuscitation	Intervention and control: Clean delivery, danger signs, emergency preparedness, facility referral. Women’s participatory groups in half of clusters	~20% of home deliveries in both study arms	A) 13195; B) 12519	ENMR	1) RR 0.95, (0.75 - 1.21)

Sibley and Sipe [60] conducted a meta-analysis in 2004 of 17 studies (n=15 286 in treatment vs 12 786 in control) and reported a 6% reduction in all-cause perinatal or neonatal deaths in the areas served by trained TBAs. TBA training was heterogeneous between studies, however, and included both primary and secondary prevention measures (neonatal resuscitation). In their pooled analysis of 3 studies (n=6217 neonates in the treatment group vs 5170 controls), TBA training was associated with an 11% reduction in “birth asphyxia” mortality, though this effect estimate also captures the effect of TBA training in neonatal resuscitation as it included 3 sites with TBA resuscitation (the SEARCH trial during the TBA training phase [62], Chandigarh, India [63], and Ethiopia [64]).

**Table 7:** GRADE summary table for the impact of traditional birth attendant training in intrapartum care on perinatal-neonatal outcomes


	**Study Quality**					**Summary of Findings**				

				**Directness**		**Endline/Intervention**		**Baseline/Control**		

**No of studies**	**Design**	**Limitations**	**Consistency**	**Generalizability to Population of Interest**	**Generalizability of intervention of interest**	**Events**	**Births**	**Events**	**Births**	**Relative Risk (95% CI)**

***Neonatal Mortality*** **(*****All Cause*****)** ***: Low outcome specific quality***										

1 [65]	Cluster RCT			Direct, rural LIC	Yes	340	9710	439	8989	aOR 0.70 (0.59-0.82)

1[74]	Cross-sectional	Low quality		Direct, rural LIC	Yes	23	909	34	119	RR 0.60 (NS)

1 [86]	Before-after	Low quality before-after, improved surveillance post		Direct, rural LIC		15	445	23	383	RR 0.66 (NS)

***Perinatal Mortality*** **(*****All Cause*****)** ***: Low outcome specific quality***										

1 [65]	Cluster RCT			Direct, rural LIC	Yes	823	9710	1077	8989	aOR 0.71 (0.62-0.83)

1 [86]	Before-after	Low quality before-after, improved surveillance post		Direct, rural LIC	Yes	99	1220	29	398	RR 0.92 (NS)

NS=Not Stated

In a Cochrane review conducted by Sibley et al [61], two studies with mortality outcomes met quality inclusion criteria. A large, cluster-randomized, controlled trial (cRCT) was conducted in Sindh, Pakistan, where TBAs in intervention areas were trained to encourage care-seeking, recognize obstetric emergencies, refer for EmOC, use clean delivery kits, and promote essential newborn care [65]. Furthermore, these TBAs were integrated into the health system by improving linkages with Lady Health Workers and community clinics. Pregnant women attended by trained TBAs were more likely to be diagnosed with obstructed labor (RR=1.26, 95% CI 1.03-1.54) and referred for EmOC (RR 1.50, 95% CI 1.19-1.90). PMR was reduced by 30% in intervention clusters (OR 0.70, 95% CI 0.60-0.80), stillbirth rate was reduced by 31% (OR 0.69, 95% CI 0.57-0.83) and NMR by 29% (OR 0.71, 95% CI 0.62-0.83). Intrapartum-related mortality was not reported; however, the concurrent reduction in both stillbirths and neonatal deaths suggests the primary prevention of intrapartum injury. The second study included in the Cochrane review was a before-after assessment of hospital-based TBA training in Guatemala [66]. Following training, there was a 53% reduction in perinatal deaths among those women referred to the hospital for delivery (16/72 pre-training vs. 24/203 post-training). However, given that the outcomes of community-based births are unknown, it was not possible to determine the impact at the population level. The two trials [65,66] were not pooled in the Cochrane analysis because of differences in study design.

Since the Cochrane evaluation [61], 3 additional trials have reported the effects of TBA training on perinatal or neonatal mortality [67,68,88] but these trials focused primarily on neonatal resuscitation and are assessed in the paper regarding neonatal resuscitation [24][88].

### Overall level of evidence

The CHERG-adapted GRADE approach and Rules for Evidence Review were applied to assess the overall quality of evidence for packages of childbirth care [17] (tables 5, 7). The quality of evidence for BEmOC or CEmOC was very low. No studies were identified of BEmOC or CEmOC as an isolated package that were usable to estimate a cause-specific neonatal mortality or an all-cause neonatal mortality effect. Nine low-grade observational studies or historical data were identified with information relevant to the effect of emergency obstetric care packages, however, these were insufficient to derive a cause-specific mortality effect. For the effect of skilled birth attendance alone on intrapartum-related neonatal deaths, 10 studies (8 observational, 2 quasi-experimental) were identified of community skilled birth attendants and there were sufficient events meeting CHERG criteria (>50) [17], however, the overall quality of evidence was low, and there were limited cause-specific mortality data. Furthermore, the studies were primarily of community midwife training, and the comparison (baseline) was a setting where skilled birth attendants already provided childbirth care, and did not reflect a counterfactual without any skilled care at birth. Therefore for all three of these intervention packages, expert opinion was obtained to derive effect estimates.

For TBA training, there were two previous meta-analyses including one cRCT. The overall level of evidence was low, and the GRADE recommendation was conditional given the limited, heterogeneous evidence, and that the intervention effectiveness is likely to be highly context specific [34,35]. Therefore no Delphi process was conducted to estimate the effect of TBAs on neonatal mortality.

### Results of Delphi process

In view of the low quality of evidence identified, a Delphi was undertaken [17]. The expert Delphi form included relevant data from the literature review (Additional File 2). A total of 21 experts participated, with representation from South Asia, Africa, Western Europe, North America, and Latin America/Caribbean. Consensus was reached in the first round for three questions (Questions 1, 2, 5), and after the 2^nd^ round for the remaining four questions (Questions 3, 4, 6, 7).

**Figure 8:**
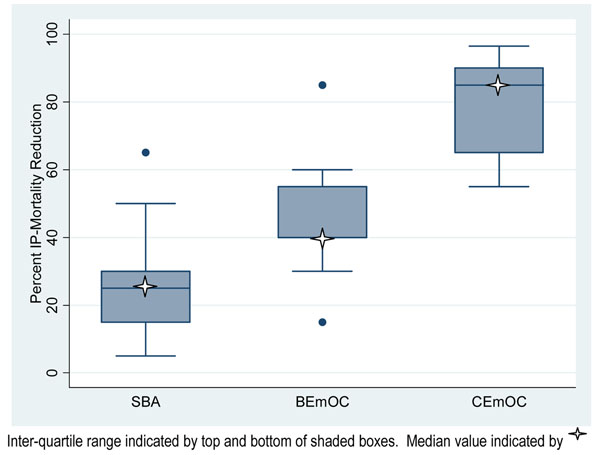
**Box plot of Delphi expert opinion effect on intrapartum-related neonatal deaths of: Skilled attendance alone, Basic Emergency Obstetric care and Comprehensive Emergency Obstetric Care (21 experts)**. Legend: Inter-quartile range indicated by top and bottom of shaded boxes. Median value indicated by

The Delphi expert panel consensus was that skilled childbirth care alone would avert 25% (range 5-65%, IQR 15-30%) of intrapartum-related neonatal deaths compared with no skilled care (figure 8). Basic and comprehensive emergency obstetric care was estimated to avert 40% (range 15-85%, IQR 40-52.5%), and 85% (range 55-96.5%, IQR 67.5-87.5%), of neonatal deaths due to intrapartum events, respectively.

## Discussion

There are 2 million deaths each year resulting from childbirth - 814,000 intrapartum related neonatal deaths, over 1 million intrapartum stillbirths and a significant proportion of the world’s 352,000 maternal deaths. Skilled childbirth care is recommended as a universal right to reduce these deaths, yet there is limited mortality evidence of the effect of childbirth care packages. The mismatch between the size of the problem and the quality of the useable evidence is stark. Our primary finding, and the main limitation of our review, is the lack of high or even moderate quality evidence of the effect of childbirth care on neonatal mortality, particularly in low and middle-income countries where the impact would be the greatest. There are a number of reasons for this low level of evidence including the challenges of ethical approval for RCTs testing care that is already considered standard, variations in obstetric packages evaluated, and inconsistencies in outcome measurement.

The variation in terminology surrounding “birth asphyxia” is a key limitation. Consistent case definitions are required; we have used the terminology “intrapartum-related” to classify neonatal deaths due to childbirth-related complications in term infants, however despite recent improvements in clarity, many of the studies identified were older and outcome definitions varied. Furthermore, in settings where the majority of neonatal deaths occur in homes, and outside of vital registration, ascertaining cause of death must often rely on verbal autopsy, which varies with respect to tools , definitions, and hierarchies used. Consistent use of such verbal autopsy tools, and more importantly the hierarchies, is critical [69]. Finally there is a paucity of data from resource-limited settings on intrapartum-related neonatal morbidity, such as neonatal encephalopathy, which requires regular neurologic assessment and is not possible for the majority of newborns in LMIC who are born at home.

The skilled birth attendance studies which we identified were heterogeneous with varying coverage and provider skill levels, and likely underestimated the effect for several reasons. First, the results for the before-after studies reflect that of *additional* midwife training, since at baseline midwives were already conducting deliveries in the community and attending deliveries, so the baseline effect is not zero. In Matlab, Bangladesh, the magnitude of the effect in the intervention vs. comparison villages was diluted by the low coverage of midwives at birth (only 25%)[50]. Furthermore, in many communities, formally trained midwives are only sought for complicated deliveries where the baby is already compromised and could only have been saved by emergency obstetric care, which may not be available.

Given the lack of cause-specific mortality evidence, we followed the LiST rules based on GRADE, and the effect of the 3 obstetric care packages was estimated using Delphi expert consensus [17]. We included a variety of experts with wide geographic representation (geographic region, low-middle and high income settings) and range of expertise and background (clinical, epidemiology, obstetrics, neonatology). Consensus was reached within an IQR of 30%. However, any expert opinion process is clearly limited, and far from ideal.

Nonetheless, the potential for major mortality reductions with skilled intrapartum care, particularly due to intrapartum-related neonatal deaths, is widely accepted and consistent with historical data from the UK, Finland[1], and Malaysia[40]. Whilst the lack of RCT evidence for the provision or non-provision of childbirth care is understandable, given that it would be unethical to conduct such trials, the dearth of observational studies of quality improvement of childbirth care assessing its effect on neonatal mortality is disappointing and a clear priority for more research. The few significant, large intervention trials of direct relevance for establishing mortality effect estimates were those of community midwife training, EmOC training, and individual interventions to improve labor monitoring and interventions (such as use of the partograph or fetal monitoring) that are reviewed in detail in two other publication supplements [10,20,21]. In some studies, there were specific missed opportunities to collect relevant perinatal outcome data. The QUARITE trial, a cluster-randomized trial of quality improvement in obstetric care via emergency obstetric care training (ALARM) and maternal death reviews, is presently underway and has perinatal-neonatal mortality as a secondary outcome [70]. This, and hopefully many more such evaluations, will help to fill a critical information gap.

For the 60 million women who deliver at home world-wide, achieving universal skilled birth attendance may require decades, and in the meantime many preventable deaths occur each year, primarily at community level [71]. TBAs attend up to 40% of births in South Asia, while the majority of home births in Africa are unattended [35]. The evidence for TBA training programs is of low quality and heterogeneous [60,61,72,73] and their role remains controversial. However one recent cRCT which emphasized partnership of TBAs with community health workers and links with the formal health system yielded promising reductions in stillbirth and neonatal mortality [65]. Early recognition of obstetric complications, including obstructed labor, and higher referral rates for emergency obstetric care were observed in this trial, and would presumably be associated with reductions in intrapartum-related injury. Several other studies have evaluated the impact of TBA training on obstetric danger sign recognition and referral [66,74] ; a meta-analysis reported a small, positive association between training and TBA referral-maternal health service utilization [72]. Given that the skills, role and training of the TBA may vary widely between regions and communities, and that the quality of evidence regarding training effectiveness is low and heterogeneous, the GRADE recommendation for implementation is presently conditional [35] and we did not attempt to estimate the effect size. However, the potential for TBAs to integrate and partner with the formal health system is promising, and requires further evaluation at scale and in varying contexts.

During the 1990s, the coverage of skilled birth attendance in Sub-Saharan Africa and South Asia increased little, but recent years have seen increases in a few countries. A contributor to the increasing coverage has included demand-side financing (eg voucher schemes or conditional cash transfers in India [75,76]), eliminating user fees (eg Ghana [77] and South Africa [78]) and the introduction of health insurance schemes (eg, Mauritania [79]), as reviewed recently [80]. Furthermore, innovative strategies to increase the supply of obstetric care have emerged, including task-shifting and the use of non-physician clinicians [10]. In Mozambique, assistant medical officers (técnicos de cirurgia) perform Caesarean section with no difference in complications or mortality rates compared to obstetricians [81,82]. Training of non-physician clinicians has been prioritized in Ethiopia, Malawi, Zambia and Mozambique, in order to fill the human resource gap. In South Asia, task shifting has involved training general practitioners, nurses and medical officers in obstetrics and anesthesia to expand coverage of EmOC [10]. Increasing the coverage of skilled obstetric care, particularly to reach the poorest, requires creative demand and supply side strategies, with sustained political and financial commitment by governments.

## Conclusion

While skilled obstetrical care is the standard of care in high income countries, the quality of evidence of the impact of childbirth care packages on intrapartum-related neonatal mortality applicable to low-income settings is low. Given the lack of epidemiologic evidence, expert opinion was used and is rated as very low quality. Our results suggest the following effectiveness on intrapartum-related neonatal deaths: 1) skilled childbirth care alone, 25%; 2) BEmOC, 40%;  3) CEmOC, 85% (table 8). Using LiST with these effect estimates, we estimate that a total of 591,000 lives of those currently dying from intrapartum related causes (“birth asphyxia”, 814,000) could be saved by providing universal access to comprehensive obstetric care. This estimate is conservative as comprehensive obstetric care would also be expected to reduce deaths from other causes of neonatal death, notably infections and preterm birth. In addition a significant proportion of maternal deaths and 1 million stillbirths could likely be saved with intrapartum interventions [32,83-85]

**Table 8:** Cause-specific mortality effect and GRADE of the estimates for obstetric care packages on intrapartum-related neonatal deaths

Effect of Comprehensive Emergency Obstetric Care *Cause specific mortality to act on:* Intrapartum related neonatal deaths *Quality of input evidence:* Very Low – effect estimates derived from Delphi panel consensus Low quality supporting evidence (8 observational, 1 quasi-experimental) *GRADE recommendation* Strong, based on clear biological mechanism *Cause specific effect and range:* Reduction in intrapartum related neonatal deaths: 85%; IQR 67.5-87.5% *Limitations of the evidence:* Evidence without cause-specific mortality effect, and with varying content of packages and varying contexts for evaluation. Only one quasi experimental design study identified
**Effect of Basic Emergency Obstetric Care** *Cause specific mortality to act on:* Intrapartum related neonatal deaths *Quality of input evidence:* Very Low – effect estimates derived from Delphi panel consensus No studies identified specifically of BEmOC with perinatal health outcomes reported *GRADE recommendation* Strong based on clear biological mechanism *Cause specific effect and range:* Reduction in intrapartum related neonatal deaths: 40%; IQR 40-52.5% *Limitations of the evidence:* No evidence available regarding effect of this specific package, even from observational designs.

**Effect of Skilled Childbirth Care** *Cause specific mortality to act on:* Intrapartum related neonatal deaths *Quality of input evidence:* Very low – effect estimates derived from Delphi panel consensus Low quality supporting evidence (2 Quasi-experimental, 8 observational) *GRADE recommendation* Strong *Cause specific effect and range:* Reduction in intrapartum related neonatal deaths: 25%; IQR 15-30% *Limitations of the evidence:* Single study with cause-specific mortality effect. For the studies identified the content of the packages tested and the contexts for evaluation and evaluation designs were variable

**Effect of Trained Traditional Birth Attendants** *Quality of input evidence:* Low quality supporting evidence (3 cRCT, 1 quasi-experimental, 5 observational) *GRADE recommendation* Conditional, dependent on local context and health system *Cause specific effect and range:* Not estimated for LiST since GRADE recommendation is conditional *Limitations of the evidence:* Supporting evidence without cause-specific mortality effect, and with varying content of packages and varying contexts for evaluation. 5 studies primarily of TBA training in neonatal resuscitation that is NOT included as part of the estimate for childbirth care package

The potential for major mortality impact emphasizes the urgent need to invest in childbirth care, improving services for those already giving birth in facilities, and reaching the 60 million women giving birth outside facilities. Roles and impact of training other cadres, such as TBAs, to link mothers with obstetric care requires further evaluation. The lack of data, even descriptive studies, to assess the effectiveness of these UN recommended packages of childbirth care highlights the need for more evaluation. Programmatic planning is required to assess the impact and cost of various packages and implementation strategies in varying contexts, and to strategize how best to close equity gaps for rural, poor families and how to close quality gaps that cost the lives of many women and babies at birth.

## List of abbreviations used

BEmOC=Basic Emergency Obstetric Care; CEmOC=Comprehensive Emergency Obstetric Care; SBA= Skilled Birth Attendant; TBA= Traditional Birth Attendant; WHO= World Health Organization; CHERG =Child Health Epidemiology Reference Group; GBD= Global Burden of Disease; PMR=perinatal mortality rate; NMR=neonatal mortality rate; ENMR =early neonatal mortality rate

## Competing interests

The authors declare that they have no competing interests.

## Authors’ contributions

ACL, JL, GLD, RH undertook the searches and abstraction. ACL, SC, JL, GLD, and HB undertook the meta analyses. ACL, JL, HB, NM, SB and GLD organised the Delphi process. ACL and JL provided the initial draft of the paper and all authors contributed. All authors read and approved the final manuscript.

## Supplementary Material

Additional file 1 is an excel sheet that contains fives sheets each of which has a table presenting extraction criteria and outputs for studies used in the meta-analysis. Click here for file

Additional File 2 is word document that contains the Delphi form used in the Delphi process and as well as background information and appendices that were provided to the Delphi participants. Click here for file
