# Supplementary material for: Care during labor and birth for the prevention of intrapartum-related neonatal deaths: a systematic review and Delphi estimation of mortality effect
Source: BMC Public Health. 2011 Apr 13;11(Suppl 3):S10. doi: 10.1186/1471-2458-11-S3-S10 (PMC3231883; doi:10.1186/1471-2458-11-S3-S10)
Supplement: Additional File 2 — is word document that contains the Delphi form used in the Delphi process and as well as background information and appendices that were provided to the Delphi participants. [file 1471-2458-11-S3-S10-S2.docx]

**Additional File 2: Delphi Form**

**Lives Saved Tool (LiST)** Expert consensus panel via a Delphi process

**Estimates of the effectiveness of care during labor and birth on cause specific neonatal mortality**

**Background to LiST**

The Lives Saved Tool (LiST) is based on *The Lancet* Child Survival and Neonatal Survival series modelling for lives saved and is now built into a widely accepted demographic software package (Spectrum^TM^). The core of Spectrum is a demographic projection model which projects the population by age and sex. LiST is a module that incorporates recent mortality rates by country and cause-of-death data for newborns and children based on definitions and estimates established by the Child Health Epidemiology Group (CHERG). The LiST tool models the impact of increasing coverage of individual interventions on the reduction of neonatal deaths by specific cause-of-death. The LiST tool does not currently include stillbirths, although this will be added soon to the model. To use the LiST tool, the user is given a menu of interventions preset with estimates of current national coverage level (eg from Demographic and Health Surveys), and then the user sets coverage targets for each intervention by year up to 2015. The increases in coverage are linked to cause-specific mortality effect estimates. The estimates of lives saved are modelled such that neonatal deaths are assigned a single cause and lives cannot be saved twice by linked interventions. A prototype of the tool can be downloaded at: [**h**ttp://www.healthpolicyinitiative.com/index.cfm?id=software&get=Spectrum](http://www.healthpolicyinitiative.com/index.cfm?id=software&get=Spectrum)

The mortality effect estimates used in the tool are based on a consistent literature review process presently being led by CHERG, using an adapted version of the WHO GRADE criteria to evaluate the quality of evidence, and conduct meta-analyses of intervention effect size where appropriate. In cases of insufficient evidence, expert opinion is being sought in order to arrive at effect estimates. The detailed technological basis, cause-specific mortality reviews and effect sizes used in the LiST tool will be published in a peer review journal supplement so that the assumptions and inputs are all in the public domain.

**Background to this Delphi process**

You have been approached to participate in this Delphi process because of your expertise in this topic. We are asking you to answer 7 questions on the following page. We will collate the responses. If there is strong consensus on the first round, a second round will not be necessary but in many cases two or even three rounds are required. To meet the deadline to be included in LiST we need to complete the estimates by last week of May and so please reply to first round by **Saturday May 23rd**

Given that it would be unethical to undertake randomized trials of obstetric interventions, the evidence for the effect on neonatal mortality is lacking. After systematic literature review, no RCTs were identified. We found 6 observational before-after or ecological studies and 1 quasi-experimental study of community skilled birth attendants that reported mortality outcomes (appendix 1). A meta-analysis was conducted of 2 before-after and 1 quasi-experimental studies, and skilled community midwives reduced early neonatal mortality by 19% (95% CI 10-26%). We identified 4 intervention studies of emergency obstetric care with either perinatal-neonatal mortality or “asphyxia” specific outcomes (appendix 2); however, all studies were of low quality and intervention packages were quite heterogeneous. Thus, we are requesting your expert opinion to estimate the impact size of obstetric care during labor and childbirth on neonatal mortality.

**Expert opinion requested from you**

**Intervention:** We are asking you to estimate the percentage of neonatal lives saved by 4 levels of packages as defined in the table on page 3, up to full comprehensive obstetric care. These packages include care provided during labor and childbirth, but the care may have been initiated during the antenatal period, eg screening for abnormal lie and decision for elective C-section; screening and management of Hypertensive Disease of Pregnancy/eclampsia; as well as management of acute intrapartum events such as APH, and obstructed labor.

This estimate does NOT include immediate postnatal interventions for the baby (warming, drying, stimulation or neonatal resuscitation with bag-and-mask). The effect of these practices for the baby would be applied as additional lives saved on the residual deaths occurring after the effect of intrapartum obstetric care has been applied. For example if you estimate that comprehensive obstetric care averts 80% of term intrapartum-related neonatal deaths, then if neonatal resuscitation with bag and mask (30% effect in model) is added the total lives saved would be 80% plus 30% of (100-80) = 80% +6% = 86%.

Also excluded are a few specific interventions which are in LiST but affect other neonatal causes of death, eg corticosteroids for preterm labor (affects preterm deaths), antibiotics for preterm PROM (affects infections deaths).

**Causes of death to act on and relevant background data**: The estimate refers to percentage reduction in each cause-specific death for each of the interventions shown in the table (page 3). In LiST, the data is preloaded for each country with the baseline number of deaths due to the following causes. Estimated mortality reduction will directly link to these causes defined using CHERG case definitions:

1. ***Intrapartum-related term neonatal deaths (previously referred to as “birth asphyxia”)*** ie term live-born babies affected by an acute obstetric emergency such as obstructed labor, pre-eclampsia etc. Some of these babies may die at birth or some may develop neonatal encephalopathy and die. This also includes a smaller group of babies dying from birth injury. **NOTE:** Intrapartum stillbirths are not yet included in LiST so should NOT be considered here.

Modelling of studies reporting incidence of Neonatal Encephalopathy (NE) in industrialized and developing countries was used in an effort to provide some more background data for an effect estimate for the comprehensive obstetric care package. These data come from systematic literature searches for the Global Burden of Disease. NE incidence rates were plotted against percent skilled delivery/facility birth proportion. A 90% reduction in NE incidence is predicted as one moves from a setting of 0% skilled birth attendance to a setting of 100% skilled birth attendance, but it should be noted that the best settings are in very well resourced high-income countries so this would be an upper limit, especially as neonatal resuscitation is included in this effect estimate.

1. ***Neonatal severe infections (sepsis, pneumonia, meningitis).*** Note that around 30% of neonatal sepsis deaths are early onset (within 72 hours after birth) and some of these are related to vaginal carriage such as Group B strep and are unlikely to be directly prevented by clean birth practices.

3) ***Neonatal tetanus.*** Note that around 40% of neonatal tetanus is thought to be related to birth practices and 60% to postnatal practices (eg cord care at home) although this can vary with specific local traditions.

**Mortality effect:** We request that you provide your best estimate of the % of neonatal deaths that could be prevented for each of these causes if the proposed intervention had been available to all who died from this cause. The comparison group you should imagine is an unattended birth. These estimates are for effectiveness in a “typical,” real-life developing country setting as opposed to efficacy under optimal conditions in a research study.

Please enter your answers in the table on the next page, and add your name (leave blank if you do not wish to be named) and send your reply back to Dr Anne CC Lee [aclee@jhsph.edu](mailto:aclee@jhsph.edu) with a copy Dr Joy Lawn [joylawn@yahoo.co.uk](mailto:joylawn@yahoo.co.uk)

**SPECIFIC QUESTIONS TO ANSWER PLEASE**

(PLEASE FILL YOUR ANSWERS ON THE MARKED SPACE IN THE TABLE BELOW)

1. If 100 babies are currently dying from **severe infection** (sepsis, meningitis, pneumonia), how many would be saved with **clean practices at home**, bearing in mind that around 30% of such deaths are early onset sepsis (ie first 3 days) and may be related to labor and birth although there are other causes of early onset sepsis eg Group B strep and vaginal carriage.
2. If 100 babies are currently dying from **severe infection** (sepsis, meningitis, pneumonia), how many would be saved with **clean practices in a facility** during labor, bearing in mind that around 30% of such deaths are early onset sepsis (ie first 3 days) and may be related to labor and birth although there are other causes of early onset sepsis eg Group B strep and vaginal carriage.
3. If 100 babies are currently dying from **neonatal tetanus** how many would be saved with **clean practices at home**, bearing in mind that around 40% of such deaths may be related to intrapartum care practices and the majority are postnatal.
4. If 100 babies are currently dying from **neonatal tetanus** how many would be saved with **clean practices in a facility** during labor, bearing in mind that around 40% of such deaths may be related to intrapartum care practices.
5. If 100 full term babies are currently dying from **intrapartum related events** (“birth asphyxia”), how many would be saved with only **skilled attendance at birth either in a primary care clinic or at home** (monitoring of labor and support, no emergency obstetric care) compared to a birth without any skilled attendance eg at home with no trained attendant. [see appendix 1 for some observation studies/historical data]
6. If 100 full term babies are currently dying from **intrapartum related events** (“birth asphyxia”), how many would be saved with only **Basic Emergency Obstetric Care** (BEmOC)(ie as per the UN definition with vacuum and episiotomy) compared to a birth without any skilled attendance eg at home with no trained attendant.
7. If 100 full term babies are currently dying from **intrapartum related events** (“birth asphyxia”), how many would be saved with only **Comprehensive Emergency Obstetric Care** (BEmOC) (ie the full package of obstetric care including C-section and blood) compared to a birth without any skilled attendance eg at home with no trained attendant. [see data from modeling on page 2 for background and table 2]

| **YOUR RESPONSES** | Estimated Impact on Neonatal Mortality reduction for.. | | |
| --- | --- | --- | --- |
| **Intervention** | Neonatal deaths due to infection (sepsis, meningitis, pneumonia) | Neonatal deaths due to tetanus | Neonatal deaths due to intrapartum related events in term infants |
| **Clean care at home during labor and birth:** clean hands, blade, surface and cord tie eg using clean delivery kit* | 1 = ___% | 3 = ___% | Not estimated |
| **Skilled care at birth:** clean delivery care, monitoring of labor, pain relief, hydration, support and basic skills for assisting delivery in cephalic presentation, including releasing a cord around the neck, delivery of shoulders, assisting a breech delivery but assuming no access to emergency obstetric care (EmOC)* | 2 = ___% | 4 = ___% | 5 = ___% |
| **Basic Emergency Obstetric Care (BEmOC):** as per UN definitions, including IV fluids and antibiotics, MgSO4 for eclampsia , episiotomy, vacuum, advanced skills for manual delivery of shoulder dystocia, breech, but assuming no access to C section or blood* | Assumed to be  the same as 2 | Assumed to be  the same as 4 | 6 = ___% |
| **Comprehensive Emergency Obstetric Care** (CEmOC): full package as per UN definitions including C-section and blood transfusion, as well as ultrasound diagnosis of placenta praevia and abnormal lie* |  |  | 7= ___% |

*Note that this mortality effect excludes the effect of resuscitation at birth or postnatal interventions to prevent or manage infections or “birth asphyxia” – see detai

# Appendix 1: Studies of Impact on Perinatal-Neonatal Mortality of Skilled Birth Attendants in the Community

| **Author** | **Years of study** | **Country** | **Setting** | **Study Design** | **Primary Intervention** | **Concurrent Interventions** | **Intervention Coverage** | **Intervention Births** | **Control/baseline Births** | **Outcomes Measured** | **Effect on outcome (95% CI)** |
| --- | --- | --- | --- | --- | --- | --- | --- | --- | --- | --- | --- |
| Ronsmans 2008 | 1975-1999 | Matlab, Bangladesh | Rural, 1987-1996 SBA at home | Before-after | Posting of midwives in villages (antenatal, intrapartum, newborn care) | strengthening referral systems, Transport to BEMOC | 25% of births attended by SBA | 19084 | 22413 | 1) IPR-NMR 2) NMR  3) ENMR  4) SBR  5) PMR | 1) 0.78 (*)  2) 0.77 (*)  3) 0.83 (0.74-0.92)  4) 0.85 (0.60-1.19)  5) 0.84 (0.78-0.90) |
| Yan 1989 | 1983-1986 | Shunyi, China | Rural Shunyi County, 7 of 29 townships | Before-after | Village doctors-midwives identify risk and either manage (external cephalic version, management of HDP) or refer mothers to county hospital | Improvement of neonatal ward in county hospital | 96% of pregnant women seen by village doctor-midwife | 2335 | 2212 | 1) PMR  2) EMR | 1) 0.66 (0.44-0.98)  2) 0.77 (0.43-1.36) |
| Ibrahim 1992 | 1985-1988 | Khartoum, Sudan | Rural, 91% home delivery | Before-after | Training and upgrading of skills of village midwives (antenatal care, monitoring in labor) | Data collection maternal-perinatal outcomes, referral system to hospital | 91% of births delivered by village midwives | 2298 | 3977 | 1) NMR  2) ENMR  3) SBR | 1) 0.68 (0.48-0.97)  2) 0.78 (0.61-1.01)  3) 0.85 (0.60-1.19) |
| Andersson 2000 | 1831-1899 | Sweden | 18 Parishes Northern Sweden | Historical | 1829 Training of midwives in use of forceps, "sharp hooks and perforators" | 1881 antiseptic techniques | 73% of home deliveries attended by midwives at endline (43% baseline) | NS | NS | 1) PMR | 1) 0.71(0.62-0.82) |
| Not combined in formal meta-analysis as different baselines, interventions and study designs, however reported range of reduction (for sig results only)  Intrapartum related NNDs - 22%  NMR – 27 - 37%  ENMR - 17 – 22%  PMR – 16 – 36%  HIE/NE – not reported | | | | | | | | | | | |

**Appendix 2. Studies Including Emergency Obstetric Care reporting Perinatal-Neonatal Mortality or “Birth Asphyxia” Outcomes**

| **Author** | **Years of study** | **Setting** | **Study Design** | **Intervention definition** | **Concurrent interventions** | **Intervention Coverage** | **Total N**  **A) Births Endline**  **B) Births Baseline** | **Outcomes** | **Effect on outcome RR (95% CI)** |
| --- | --- | --- | --- | --- | --- | --- | --- | --- | --- |
| Edmond 2002 | 1995-1998 | Natal, Community in Northeast Brazil | Observational before-after | Opening of primary maternity facilities (BEmOC) at poly-clinic to serve low risk deliveries in the community | Antenatal care, booking of high risk pregnancies at Maternity hospital (CEmOC); community health agents conducting home visits, training in community health clinics | Deliveries at low-risk maternity clinics increased from 0% to 51% at end of period | A) 536  B) 679 | 1) Early neonatal mortality (first 7 days)  2) Stillbirth | 1) RR 0.12 (0.04-0.40)  2) RR 0.66 (0.47-0.94) |
| Draycott 2006 | 1998-2003 | South Mead Tertiary Care Hospital, UK | Observational before-after | EOC training course: CTG interpretation, course of action, obstetric emergency drills (dystocia, PPH, eclampsia, twins, breech, resuscitation) |  | Mandatory course for all midwives | A) 11030  B) 8430 | 1) Apgar at 5 min <7;  2) HIE (MacLennan): acidosis, pH<7 with hypoxic event immediately before birth-labor, sustained drop in FHR, Apgar <7 at 5min, multisystem involvement/ abnormal brain imaging | 1) RR 0.51 (0.35-0.74)  2) RR 0.50 (0.26-0.95) |
| Korhonen 1994 | 1986-1991 | Maternity Hospital; Helsinki, Finland | Prospective Observational | Emergency Caesarean Team in Hospital vs. on call (out of hospital, 10 minute average delay) |  | NS | A) 60  B) 41 | 1) Fetal Death;  2) Hypoxic Ischemic Encephalopathy | 1) 3 in utero fetal deaths in control (on-call) group  2) 1 case of HIE in control (on-call) group |
| Koblinsky 1999 | 1957-1990s | Malaysia - National | Historical-ecological | 1960s Training of professional village midwives, linking to regional clinics, referral to district hospitals; 1980's shift to facility births | 3 decades of perinatal care and obstetric care upgrading, as well as child health interventions | By 1996, 95% of home births by midwives; by 1998 90% of women with high risk, 80% moderate risk delivering in hospitals | Not reported | 1) NMR | NMR from 75.5 (1957) to 14.8 (1991) |
| Not combined in formal meta-analysis as different baselines, multiple concurrent interventions, and varying evaluation designs however reported range of reduction (for sig results only)  NMR ~80%  ENMR 88%  SBR and 40-40%  HIE/NE 50% reduction | | | | | | | | | |
